# Resveratrol prevents nanoparticles-induced inflammation and oxidative stress via downregulation of PKC-α and NADPH oxidase in lung epithelial A549 cells

**DOI:** 10.1186/s12906-018-2278-6

**Published:** 2018-07-09

**Authors:** Hung-Te Hsu, Yu-Ting Tseng, Wen-Jhe Wong, Chi-Ming Liu, Yi-Ching Lo

**Affiliations:** 1 Faculty of Anesthesiology, School of Medicine, College of Medicine, Kaohsiung Medical University, Kaohsiung, Taiwan; 2 Department of Anesthesia, Kaohsiung Municipal Ta-Tung Hospital, Kaohsiung, Taiwan; 3 Department of Pharmacology, School of Medicine, College of Medicine, Kaohsiung Medical University, 100 Shih-Chuan 1st Road, Kaohsiung, 80708 Taiwan; 4 Department of Nursing, Tzu Hui Institute of Technology, Pingtung County, 92641 Taiwan

**Keywords:** Nanoparticles, Resveratrol, PKC-α, NADPH oxidase, Inflammation, Oxidative stress

## Abstract

**Background:**

Exposure to carbon black nanoparticles (CBNPs), a well-known industrial production, promotes pulmonary toxicity through inflammation and oxidative stress. Recent studies show that some polyphenols exert their antioxidant properties through regulation of protein kinase C-α (PKC-α) and NADPH oxidase (Nox) signaling. Resveratrol, a dietary polyphenol in fruits, possesses various health beneficial effects including anti-inflammatory and antioxidative properties. In this study, we aimed to elucidate the involvement of PKC-α and Nox in CBNPs-induced inflammation and oxidative stress, and to investigate the protective effects of resveratrol on CBNP-induced inflammation and oxidative stress in human lung epithelial A549 cells.

**Methods:**

The production of reactive oxygen species (ROS) and the change of mitochondrial membrane potential (ΔΨm) were measured by flow cytometry. Nitric oxide (NO) was measured using the Griess reagent, and prostaglandin E_2_ (PGE_2_) production was detected by ELISA, while protein expressions were measured by Western blotting analysis.

**Results:**

In lung epithelial A549 cells, CBNPs significantly enhanced oxidative stress by upregulation of Nox2 and membrane expression of p67^phox^ accompanied with increase of ROS production. CBNPs also increased inflammatory factors, including iNOS, COX-2, NO and PGE_2_. However, resveratrol attenuated the above effects induced by CBNPs in A549 cells; additionally, CBNPs-induced activation of PKC-α was observed. We found that PKC-α inhibitor (Gö6976) could attenuate CBNPs-induced inflammation by down-regulation of ROS, NO and PGE_2_ production in A549 cells, suggesting PKC-α might be involved in CBNPs-induced oxidative stress and inflammation. Our results also found resveratrol was able to inhibit protein expression of PKC-α induced by CBNPs. Moreover, ROS scavenger (NAC) and Nox inhibitor (DPI) attenuated CBNPs-induced expressions of iNOS and COX-2. DPI could also attenuate CBNPs-induced ROS, NO and PGE_2_ production.

**Conclusions:**

Resveratrol attenuated CBNPs-induced oxidative and inflammatory factors in lung epithelial A549 cells, at least in part via inhibiting PKC-α- and Nox-related signaling.

## Background

Nanoparticles (NPs) are defined as particles with any external dimension, internal structure, or surface structure in the nanoscale, approximately 1 to 100 nm [1]. NPs have been widely used in various commercial products such as medicine, cosmetic, and biotechnology etc., and can be found in many different environmental exposures. NPs-induced inflammation is associated with multiple diverse cardiopulmonary diseases because of their unique toxic properties. The NPs can easily enter the lungs during respiration due to their tiny size with a diameter less than 100 nm [2], subsequently inducing apoptosis and pro-inflammatory reaction in lung epithelial cells [3].

Carbon black nanoparticles (CBNPs) are one of the most used nanomaterials, which can interact with various biological systems and affect specific cellular function [4–6]. Till now, research on assessment of human exposure to CBNPs is scant. However, while CBNPs act as a core component of many ultrafine pollutants, the general public exposure is unpredictable. CBNPs can damage different organ systems by different exposure routes, and pulmonary inhalation is known as a major route of CBNPs exposure as CBNPs can impair the respiratory system leading to lung inflammation and fibrosis [7–9]. Exposure to CBNPs is known to induce lung inflammation in vivo [10, 11], and possesses the potential to generate reactive oxygen species (ROS) and inflammatory factors [12–16]. It has been suggested that CBNPs induce apoptosis by a ROS-dependent mitochondrial pathway in bronchial epithelial cells [13], and induce inflammatory response through ROS-NFκB pathway in macrophages [14]. Also, ERK MAP kinase and p38 MAP kinase might be involved in CBNPs-induced ROS production in alveolar macrophage [12].

Protein kinase C (PKC) activation has been identified to play a critical role in inflammation and oxidative stress. The PKC-α suppression also inhibited MMP-9 and COX-2 expression in human monocytes [17]. An enzyme which is well-known to be involved in signal transduction associated with inflammation and oxidative stress is the NADPH oxidase (Nox). A crosstalk between PKC and Nox has been reported for instance, in human airway epithelial cells, where PKC activation induced Nox-dependent ROS production [18]. In rat brain astrocytes, it has also been observed that bradykinin-induced inflammation via activation of PKC-α mediated Nox2/ROS signaling [19]. Recently, the NPs-induced Nox2 activation in macrophages has been reported [20]. Lung epithelial cells in the respiratory tract are the first barrier in contact with these inhalable NPs, and A549 human lung epithelial cells have been wildly used for studying NPs-induced cytotoxicity [21–23]; accordingly, in this study, we further investigated the role of PKC-α and Nox activation in CBNPs-induced inflammation on A549 cells.

A growing amount of evidence has demonstrated that inflammatory processes and particle-associated oxidative stress play major roles in particle-induced diseases. Such evidence is addressing a growing interest toward anti-inflammatory agents and anti-oxidants as preventive or curative treatments of environmentally-induced lung inflammation. Therefore, in this study, a well-known anti-inflammatory and anti-oxidative agent, resveratrol, was examined for its protective effects against nanoparticle toxicity. Resveratrol is a dietary polyphenol in fruits and food products that blocks the action of LPS-induced inflammatory mediators, including NO, PGE_2_, TNF-α, and IL-1β through PI3K/Akt activation in RAW264.7 cells [24] and decreases cigarette smoke-induced ROS production in keratinocytes [25]. The protective effects and mechanisms of resveratrol on CBNPs-induced inflammation and ROS production were evaluated in this study.

The present study highlights the molecular mechanism of CBNPs-activated PKC-α and Nox linking the NPs-induced oxidative stress and inflammation, exploring the preventive molecular strategy against NPs-induced inflammation**.**

## Methods

### Materials

Resveratrol, bovine serum albumin (BSA), apocynin, diphenylene iodonium (DPI), N-acetyl cysteine (NAC), L-NG-Nitroarginine Methyl Ester (L-NAME), dimethyl sulfoxide (DMSO), 2′,7′-dichloro-dihydrofluorescein diacetate (H_2_DCF-DA), 12-(2-Cyanoethyl)-6,7,12,13-tetrahydro-13-methyl-5-oxo-5H-indolo(2,3-a)pyrrolo(3,4-c)-carbazole (Gö6976), (+)-5-methyl-10,11-dihydro-5H-dibenzo [a,d] cyclohepten-5,10-imine maleate (MK-801), verapamil, 3-(4,5-dimethylthiazol- 2-yl)-2,5-diphenyltetrazolium bromide (MTT), Hoechst 33,342, Triton X-100, and mouse antibody against β-actin and iNOS were obtained from Sigma–Aldrich (St. Louis, MO, USA). Dulbecco’s modified Eagle’s medium (DMEM), fetal bovine serum (FBS), penicillin, amphotericin B, streptomycin, trypsin-EDTA, and 5,5′,6,6′-tetrachloro- 1,1′,3,3′-tetraethyl benzimidazolylcarbocyanine iodide (JC-1) were obtained from Invitrogen (Carlsbad, CA, USA). All materials for SDS–PAGE were obtained from Bio-Rad (Hercules, CA, USA). NS-398, mouse antibodies against COX-2, rabbit antibody against PKC-α, p67^phox^, and all horseradish peroxidase-conjugated secondary antibodies were obtained from Santa Cruz Biotechnology (Santa Cruz, CA, USA). Mouse antibody against Nox2 was obtained from BD Bioscience (San Jose, CA, USA). Enhanced chemiluminescence reagent and polyvinylidene difluoride (PVDF) membranes were purchased from PerkinElmer Life and Analytical Sciences (Boston, MA, USA). The LDH cytotoxicity assay kit was purchased from G-Biosciences (St. Louis, MO, USA).

### Particle preparation

The CBNPs (Printex90®, 14 nm) were obtained from Evonik Industries/Degussa (Frankfurt, Germany). Stock suspensions of the CBNPs were made at a concentration of 1 mg/mL in DMEM, sonicated for 30 min before used.

### Cell culture

Human epithelial carcinoma cell line (A549) was purchase from Food Industry Research and Development Institute (Hsin-Chu, Taiwan). Cells were cultured in DMEM containing 10% (*v*/v) heat-inactivated FBS, 2 mM glutamine, 100 U/mL penicillin, 100 mg/mL streptomycin, and 0.25 mg/mL amphotericin B at 37 °C in a humidified incubator under 5% CO_2_ and 95% air.

### Drug treatment

To investigate the effects of CBNPs on A549 cells, cells were treated with CBNPs (1–100 μg/mL) for 24 h. To investigate the mechanisms of CBNPs-induced inflammation and oxidative stress, cells were pre-treated with verapamil (100 μM), MK801 (100 μM), Gö6976 (10 μM), DPI (10 μM), apocynin (500 μM), NAC (5 mM), L-NAME (50 μM), or NS398 (1 μM) for 1 h before addition of CBNPs (25 μg/mL) for 24 h. To investigate the protective effects of resveratrol in CBNPs-treated A549 cells, cells were pre-treated with resveratrol (1, 5, and 10 μM) for 1 h before addition of CBNPs (25 μg/mL) for 24 h. The control group in this study were cells without any treatment.

### Determination of cell viability

The cell viability was determined by MTT (3-(4,5-Dimethylthiazol-2-yl)-2,5-diphenyl tetrazolium bromide) assay and lactate dehydrogenase (LDH) assay as previous described [26]. The principle of MTT assay is based on the cleavage of tetrazolium ring of MTT by dehydrogenases in active mitochondria of living cells as an estimate on cell viability. In MTT assay, cells were treated with CBNPs (1–100 μg/mL) for 24 h or pre-treated with resveratrol (1, 5, and 10 μM) for 1 h before addition of CBNPs (25 μg/mL) for 24 h, the medium was replaced with MTT at a final concentration of 0.5 mg/mL for 3 h at 37 °C and 5% CO_2_. The formazan crystals in the cells were solubilized with 100 μL DMSO. Absorbance was read at 560 nm on a microplate reader. Besides, LDH is a soluble cytosolic enzyme in cells that can be released into culture medium when cell death occurs; therefore, the presence of this enzyme in the culture medium can be used as a cell death marker. In LDH assay, culture medium was collected and assayed for LDH activity using a cytotoxicity detection kit. Briefly, the release of LDH was measured with a coupled enzymatic reaction that results in the conversion of a tetrazolium salt into red-colored formazan. The amount of formazan formed correlated with LDH activity. The formazan product was measured with a microplate reader at 490 nm.

### Measurement of mitochondrial membrane potential (ΔΨm) and reactive oxygen species (ROS) production

The change in ΔΨm was evaluated by JC-1 fluorescence staining assay [27] which was determined by the ratio between red and green fluorescence. The red JC-1 fluorescence were detected by excitation/emission at 540/570 nm, and green JC-1 fluorescence were detected by excitation/emission at 495/520 nm by Coulter CyFlow Cytometer (Partec, Germany). The production of ROS was detected by fluorescence assay that involved H_2_DCF-DA staining [28]. After 24 h of drug treatments, the A549 cells were stained with 10 μM H_2_DCF-DA at 37 °C. After 30 min of incubation, the A549 cells were detached by trypsin-EDTA and washed with PBS. One hundred thousand (1 × 10^5^) cells were analyzed and the DCF fluorescence was detected by excitation at 495 nm and emission of 520 nm by Coulter CyFlow Cytometer (Partec, Germany).

### Measurement of nitric oxide and prostaglandin E_2_

Nitrite generation was an indicator of nitric oxide (NO) release measured using the Griess reagent (1% sulfanilamide and 0.1% N-1-naphthylethylenediamide in 5% phosphoric acid) [29]. Briefly, the medium from treated cells was collected. and 50 μL was incubated for 10 min at room temperature under dark conditions with 50 μL of Griess reagent. The absorbance was measured at 540 nm (OD540) and the concentration was calculated using a sodium nitrite standard reference curve [29]. The PGE_2_ production in the supernatant was detected using EIA kit (Cayman Chemical, USA), following the manufacturer’s guide [30]. Supernatant was collected after 24 h of CBNPs (1, 10, or 25 μg/mL) treatment with or without Gö6976 (10 μM), DPI (10 μM) or resveratrol (1, 5, or 10 μM) pretreatment, and the absorbance measured at 405 nm (OD405).

### Immunocytochemistry

After 24 h of CBNPs exposure, cells were washed with PBS and fixed with 4% paraformaldehyde for 30 min, and further incubated in a permeabilizing solution (0.1% Triton X-100) for 5 min. Cells were then stained with 5 μg/mL Hoechst 33,342 for 10 min to detect DNA condensation, and nuclear fragmentation, which were features of apoptotic cells [31]. The cells were observed under fluorescence microscopy (Nikon, Japan).

### Western blotting analysis

Western blotting analysis was used to determine protein expressions. After CBNPs (1, 10, or 25 μg/mL) treatment with or without verapamil (100 μM), MK801 (100 μM), Gö6976 (10 μM), DPI (10 μM), apocynin (500 μM), NAC (5 mM), L-NAME (50 μM), NS398 (1 μM), or resveratrol (1, 5, or 10 μM) pretreatment, cytosolic or membrane protein extracts of A549 cells were collected by using lysis buffer (Thermo Scientific, Waltham, MA, USA) or commercial kit (BioVision, Mountain View, CA, USA) respectively as described previously [30]. The lysates were centrifuged at 15000×g for 30 min at 4 °C and the supernatant containing proteins was collected. The proteins were reversed by SDS-polyacrylamide gel electrophoresis after using the Bio-Rad protein assay kit to determine the total protein concentration, then the protein bands were transferred onto polyvinylidene difluoride (PVDF) membranes and incubated with TBST (50 mM Tris–HCl, pH 7.6, 150 mM NaCl, 0.1% Tween 20) containing 5% non-fat milk for 1 h at room temperature to block the non-specific binding sites. Following that, the PVDF membranes were incubated overnight at 4 °C with one of the following specific primary antibodies: rabbit anti-PKC-α (1: 1000), rabbit anti-Nox2 (1: 1000), rabbit anti-p67^phox^ (1: 1000), mouse anti-iNOS (1:500), goat anti-COX-2 (1: 1000), and mouse anti-β-actin (1: 10,000). After six wash cycles with TBST at 5 min intervals, membranes were incubated with one of the following secondary antibodies: goat anti-rabbit IgG-HRP (1: 1000), goat anti-mouse IgG HRP (1: 1000), or donkey anti-goat IgG HRP (1: 5000) for 1 h at room temperature. After six wash cycles with TBST, the protein bands were stained with the enhanced chemiluminescence reagent.

### Statistical analysis

Data are shown as the mean ± standard error of the mean (S.E.M) from six independent experiments (*n* = 6). A one-way analysis of variance (ANOVA) followed by Tukey’s test was used for all pair comparisons. A value of *P* < 0.05 was considered as statistically significant. Data were analyzed with the Statistical Package for Social Sciences (SPSS, Chicago, IL, USA).

## Results

### CBNPs decrease cell viability with loss of mitochondrial membrane potential (ΔΨm) and increase of nuclear condensation in A549 cells

**Fig. 1:**
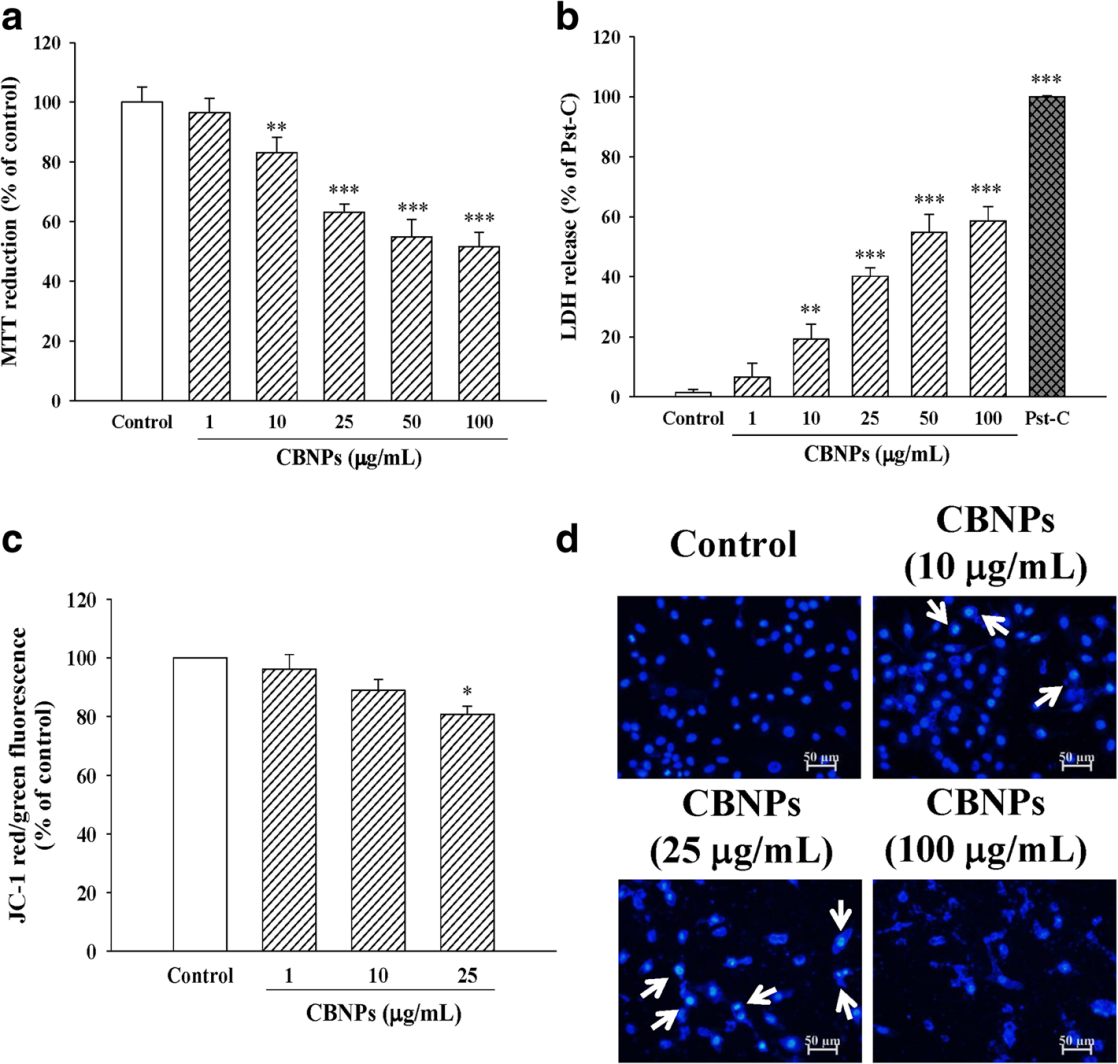
Effects of CBNPs on cell viability assessed by MTT (**a**) and LDH (**b)** assay, and on changes of mitochondrial membrane potential (ΔΨm) (**c**) and nuclear condensation (**d**) in A549 cells. ΔΨm was measured by JC-1 staining and analyzed by a flow cytometer. Nuclear condensation (white arrow) was determined by Hoechst 33,342 and observed by a fluorescent microscope. Cells were treated with CBNPs for 24 h. Bars represent the mean ± S.E.M. from six independent experiments. ^*^P < 0.05, ^**^P < 0.01, ^***^P < 0.001 vs. control group (without any treatment). Scale bar = 50 μm

To examine the cytotoxic effects of CBNPs on lung epithelial cells, cultured A549 were treated with various concentrations of CBNPs for 24 h. Results from MTT (Fig. 1a) and LDH (Fig. 1b) assay indicated that CBNPs (10–100 μg/mL) significantly decreased cell viability of A549 cells. CBNPs also decreased ΔΨm of A549 cells (Fig. 1c). Moreover, CBNPs-induced nuclear condensation of A549 cells was observed by using Hoechst 33,342 staining (Fig. 1d).

### CBNPs activate PKC-α/Nox pathway in A549 cells

**Fig. 2:**
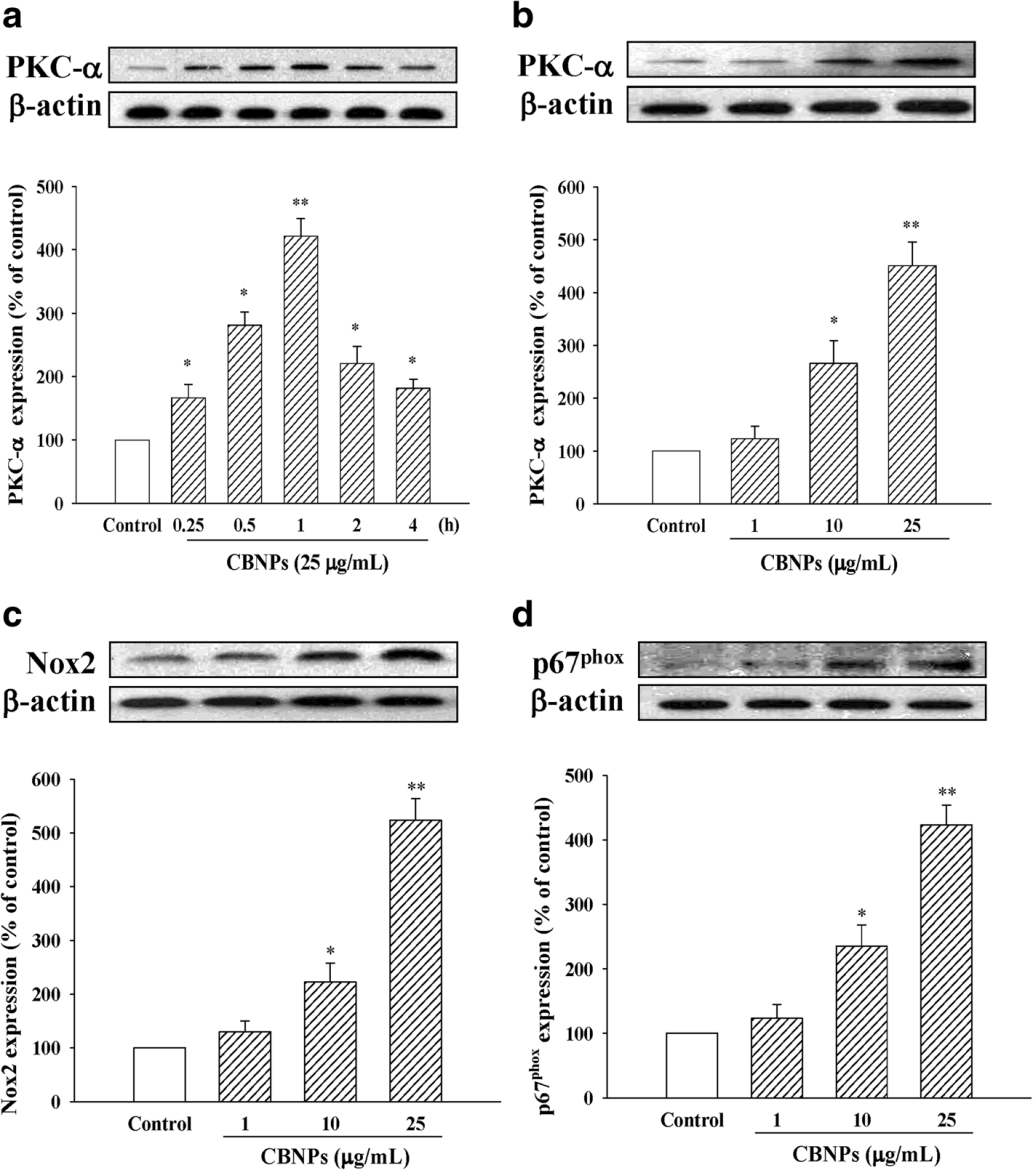
Effects of CBNPs (25 μg/mL) on PKC-α expression after 0.25 to 4 h of CBNPs treatment in A549 cells (**a**). Effects of CBNPs (1–25 μg/mL) on PKC-α expression after 1 h of CBNPs treatment in A549 cells (**b**). Effects of CBNPs on protein expression of Nox2 (**c**), membrane protein expression of p67^phox^ (**d**). **c-d** Cells were treated with CBNPs (1, 10, 25 μg/mL) for 24 h. Bars represent the mean ± S.E.M. from six independent experiments. ^*^P < 0.05, ^**^P < 0.01 vs. control group (without any treatment)

Next, we examined the effects of CBNPs on PKC-α and Nox activation. As shown in Fig. 2a, CBNPs produced its peak effect on PKC-α activation at 1 h (421.33 ± 27.75% of control). Accordingly, cells were incubated with different concentrations (1, 10 and 25 μg/mL) of CBNPs for 1 h, and the results suggested that CBNPs induced PKC-α activation occurred in a concentration-dependent manner (Fig. 2b). Also, CBNPs (10 and 25 μg/mL) enhanced epithelial Nox2 expression (Fig. 2c) and induced p67^phox^ membrane translocation (Fig. 2d).

### Roles of PKC-α and Nox on CBNPs-induced inflammation and oxidative stress

**Fig. 3:**
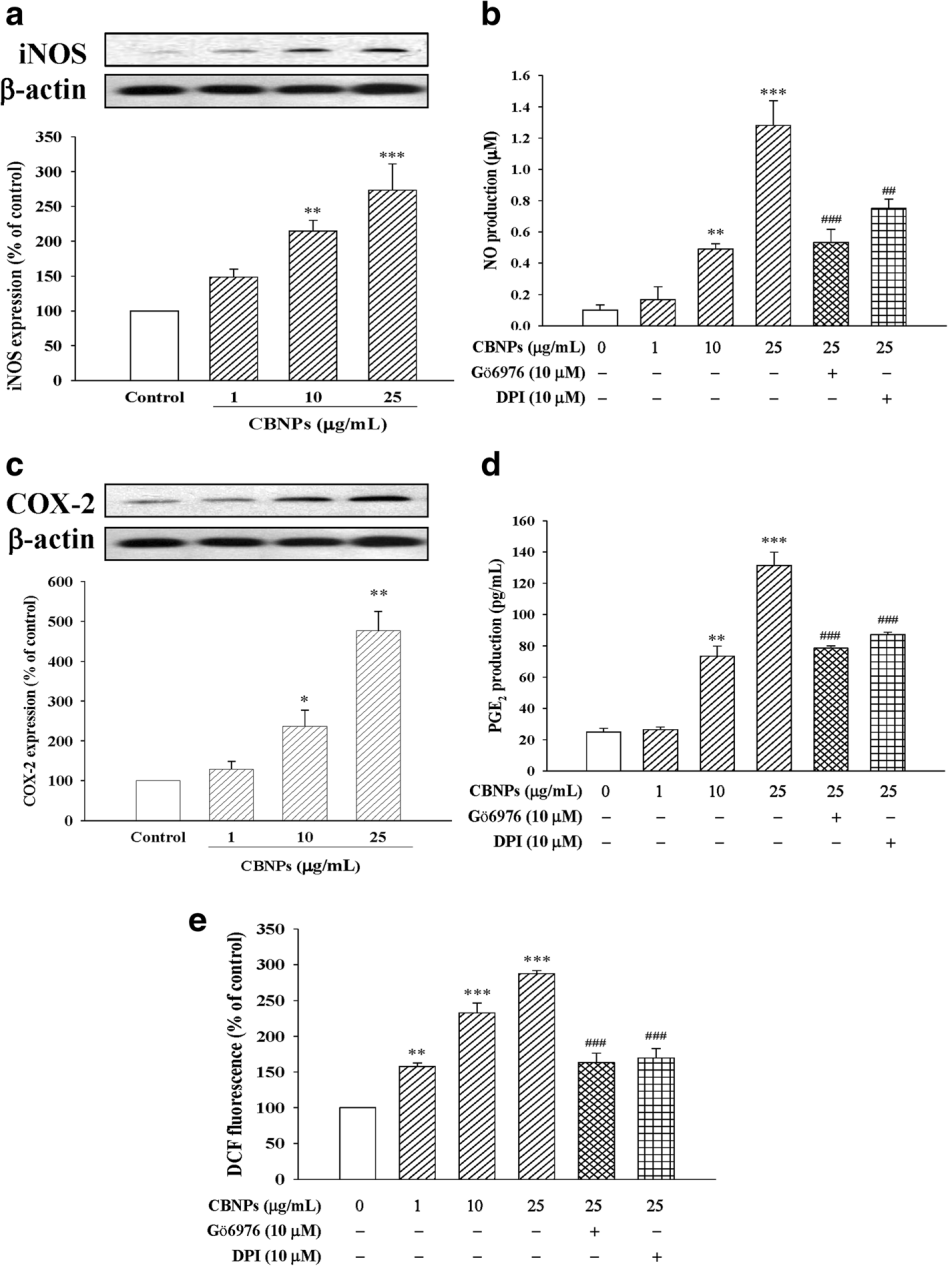
Effects of CBNPs on iNOS expression (**a**), effects of Gö6976 or DPI on NO production (**b**), COX-2 expression (**c**), and effects of Gö6976 or DPI on PGE_2_ production (**d**), and effects of Gö6976 or DPI on ROS production (**e**) in A549 cells. Cells were treated with CBNPs (1, 10, 25 μg/mL) for 24 h or pretreated with Gö6976 or DPI for 1 h respectively, and then treated with CBNPs (1–25 μg/mL) for 24 h. ROS production was measured using H_2_DCF-DA staining and detected the DCF fluorescence by flow cytometer. Bars represent the mean ± S.E.M. from six independent experiments. ^*^P < 0.05, ^**^P < 0.01, ^***^P < 0.001 vs. control group (without any treatment). ^*##*^*P* < 0.01, ^*###*^*P* < 0.001 vs. CBNPs (25 μg/mL) group

As shown in Fig. 3, CBNPs up-regulated inflammatory protein expression of iNOS (Fig. 3a) and COX-2 (Fig. 3c), and increased their downstream products NO (Fig. 3b) and PGE_2_ (Fig. 3d) respectively. The CBNPs-induced ROS production was also measured by using H_2_DCF-DA staining and detecting the DCF fluorescence. Results indicated that CBNPs significantly increased epithelial ROS production (Fig. 3e). We further examined the role of PKC-α and Nox on CBNPs-induced inflammation and oxidative stress via measuring NO, PGE_2_, and ROS production. Results demonstrated that both PKC-α inhibitor Gö6976 (10 μM) and Nox inhibitor DPI (10 μM) attenuated CBNPs-induced production of NO (Fig. 3b) and PGE_2_ (Fig. 3d). CBNPs-induced production of ROS was also inhibited by Gö6976 and DPI pretreatment (Fig. 3e).

### Roles of PKC-α on CBNPs-induced Nox activation

**Fig. 4:**
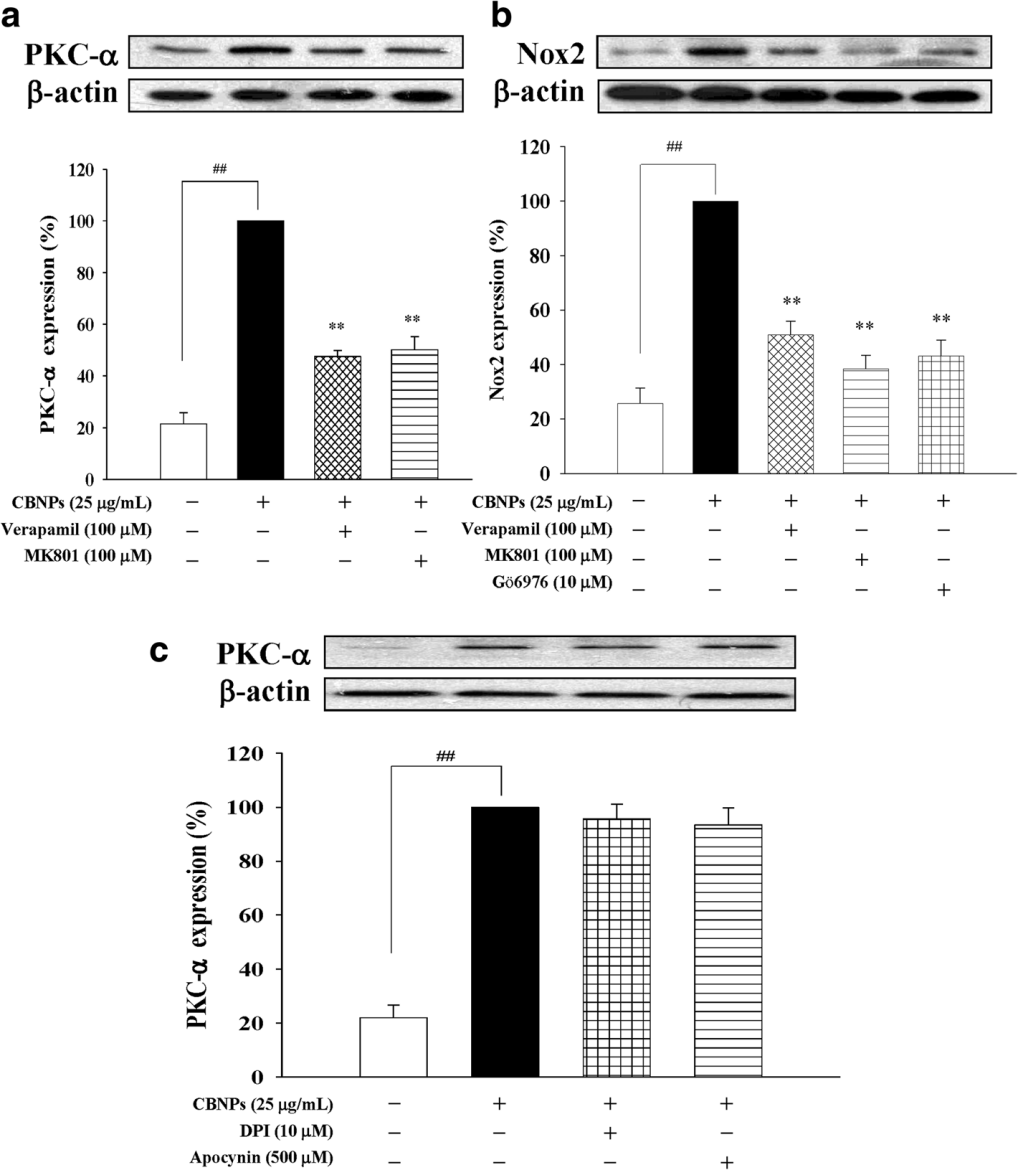
**a** Effects of verapamil or MK801 on CBNPs-induced PKC-α activation in A549 cells. **b** Effects of verapamil, MK801, or Gö6976 on CBNP-induced Nox2 activation in A549 cells. **c** Effects of DPI or apocynin on CBNPs-induced PKC-α activation in A549 cells. Cell were pretreated with verapamil, MK801, Gö69761, DPI, or apocynin for 1 h, respectively, and then treated with CBNPs (25 μg/mL) for 1 h or 24 h for determination of PKC-α and Nox, respectively. Bars represent the mean ± S.E.M. from six independent experiments. ^*##*^*P* < 0.01 vs. control group (without any treatment). ^**^P < 0.01 vs. CBNPs (25 μg/mL) group

To clarify the role of PKC-α on CBNPs-induced Nox activation, L-type Ca^2+^ channel blocker verapamil, and NMDA receptor antagonist MK-801 were added to A549 cells 1 h before CBNPs (25 μg/mL) treatment. Results indicated that both verapamil and MK-801 treatments attenuated PKC-α expression induced by CBNPs (Fig. 4a). Moreover, PKC-α inhibition (by verapamil, MK-801, and Gö6976) attenuated CBNPs-induced Nox2 expression (Fig. 4b). However, Nox inhibitors (DPI and apocynin) could not attenuate PKC-α activation caused by CBNPs (Fig. 4c).

### Roles of Nox activation and ROS production in CBNPs-induces inflammation

**Fig. 5:**
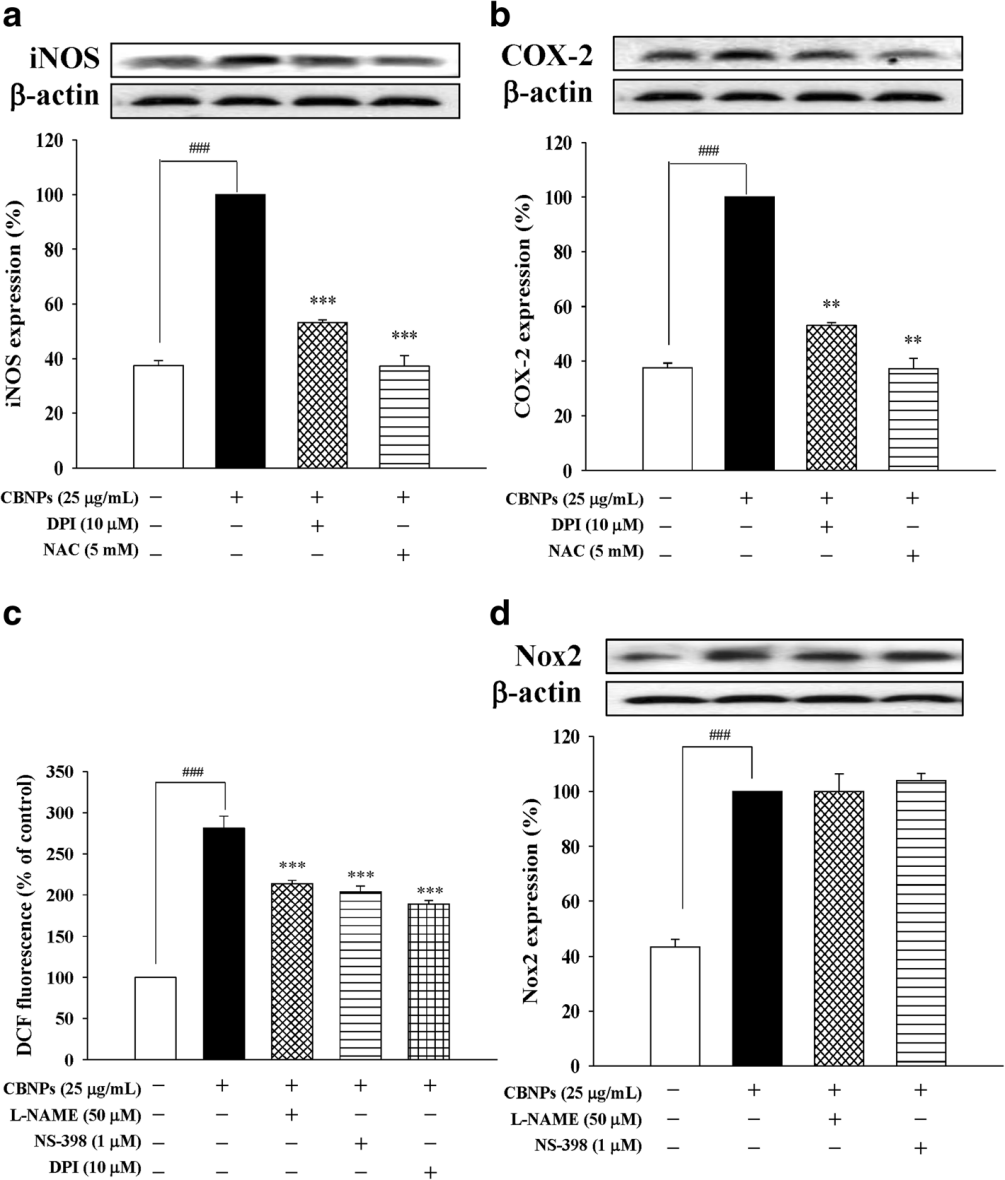
Effects of DPI or NAC on CBNP-induced iNOS (**a**) and COX-2 (**b**) expression in A549 cells. **c** Effects of L-NAME, NS-398, or DPI on CBNP-induced ROS production in A549 cells. **d** Effects of L-NAME or NS-398 on CBNP-induced Nox2 activation in A549 cells. Cells were pretreated with DPI, NAC, L-NAME, or NS-398 1 h before addition of CBNPs (25 μg/mL) for 24 h. ROS production was measured using H_2_DCF-DA staining and detected the DCF fluorescence by flow cytometer. Bars represent the mean ± S.E.M. from six independent experiments. ^*###*^*P* < 0.001 vs. control group (without any treatment). ^**^P < 0.01, ^***^P < 0.001 vs. CBNPs (25 μg/mL) group

To examine the roles of Nox and ROS on CBNPs-induced inflammation, Nox inhibitor DPI and ROS scavenger NAC were used. The present results indicated that DPI (10 μM) could attenuate CBNPs-induced expression of iNOS (Fig. 5a) and COX-2 (Fig. 5b). Similarly, CBNPs-induced expression of iNOS and COX-2 could be attenuated by NAC (5 mM) pretreatment (Figs. 5a and b). Moreover, CBNPs-induced ROS production could be attenuated by DPI, L-NAME (NOS inhibitor) and NS-398 (COX-2 inhibitor) (Fig. 5c). However, L-NAME and NS-398 could not significantly attenuate CBNPs-induced Nox2 activation (Fig. 5d).

### Resveratrol reduces CBNPs-induced cell death in A549 cells

**Fig. 6:**
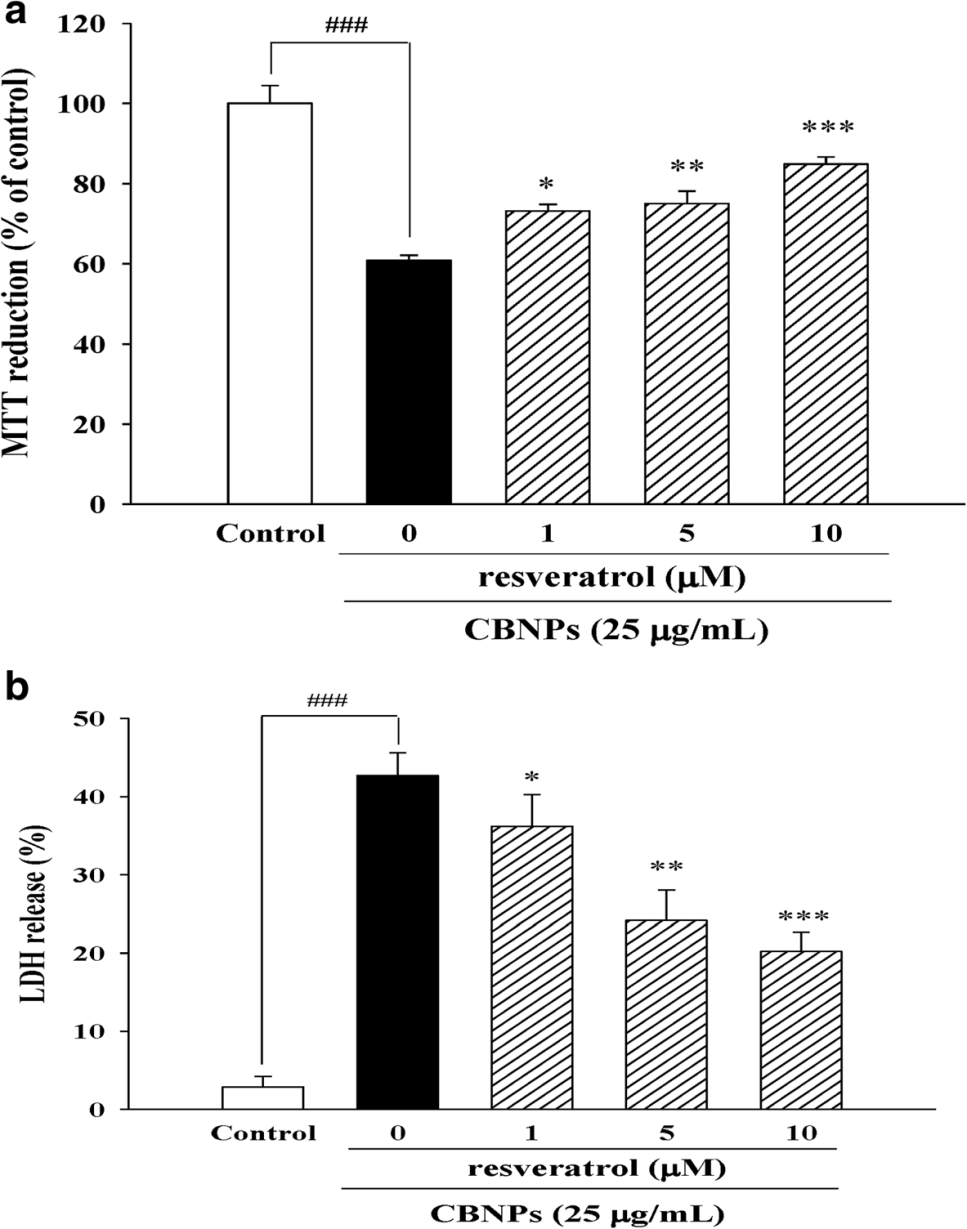
Effects of resveratrol on cell viability in CBNPs-treated A549 cells. Cells were pre-treated with resveratrol for 1 h before addition of CBNPs (25 μg/mL) for 24 h. Cell viability was measured by MTT (**a**) and LDH assay (**b**). Bars represent the mean ± S.E.M. from six independent experiments. ^*###*^*P* < 0.001 vs. control group (without any treatment). ^*^P < 0.05, ^**^P < 0.01, ^***^P < 0.001 vs. CBNPs (25 μg/mL) group

Further, we investigated the potential protective effects of the known anti-inflammatory and anti-oxidative agent, resveratrol, on CBNPs-induced epithelial cytotoxicity. A549 cells were treated with resveratrol for 1 h before the addition of CBNPs (25 μg/mL) for 24 h. Results from MTT and LDH assays indicated that resveratrol decreased cytotoxicity induced by CBNPs (Figs.6a and b).

### Resveratrol protects A549 cells against CBNPs-induced inflammation in A549 cells

**Fig. 7:**
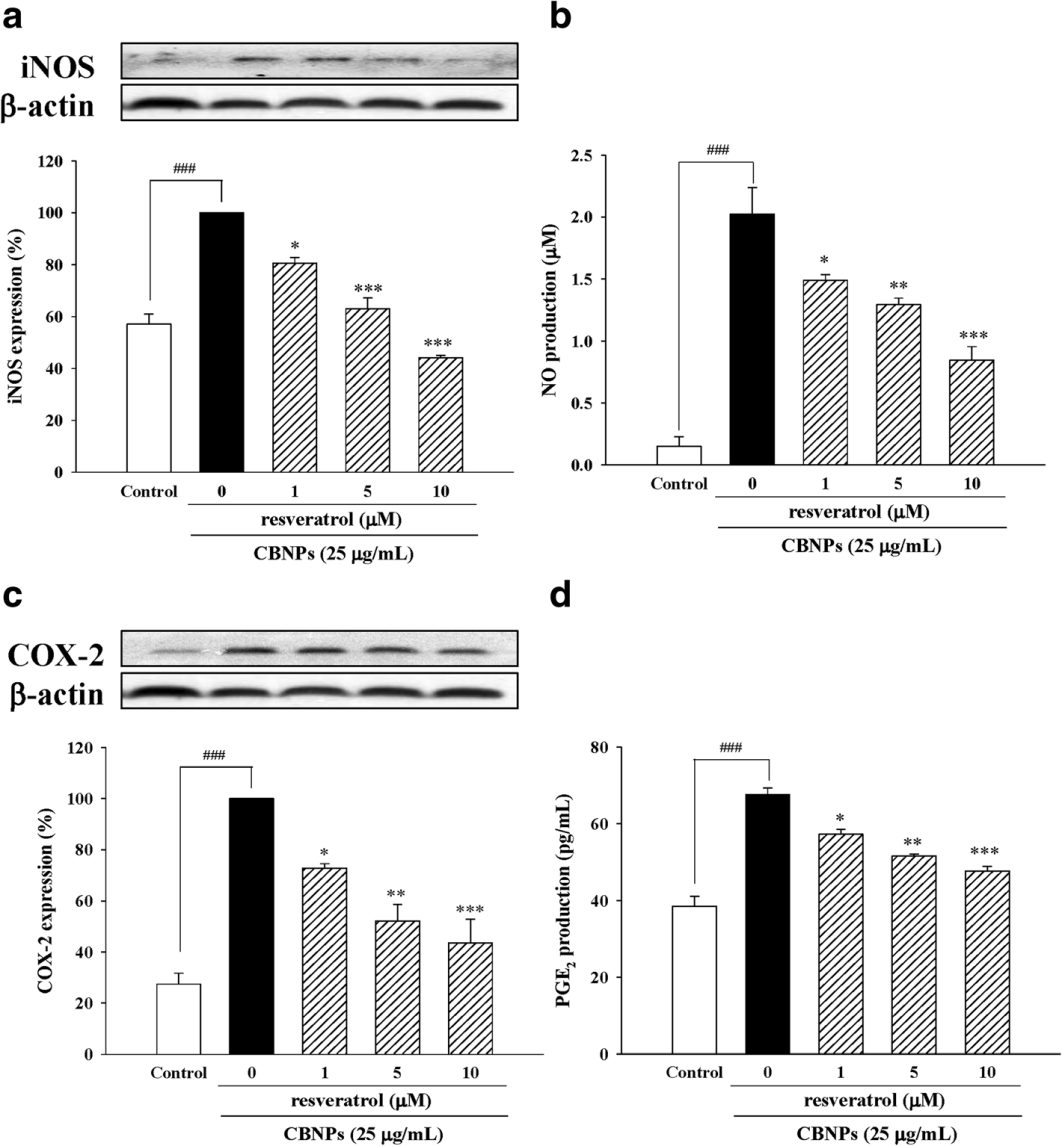
Effects of resveratrol on iNOS expression (**a**), NO production (**b**), COX-2 expression (**c**), and PGE_2_ production (**d**) in CBNPs-treated A549 cells. Cells were pre-treated with resveratrol for 1 h before addition of CBNPs (25 μg/mL) for 24 h. Bars represent the mean ± S.E.M. from six independent experiments. ^*###*^*P* < 0.001 vs. control group (without any treatment). ^*^P < 0.05, ^**^P < 0.01, ^***^P < 0.001 vs. CBNPs (25 μg/mL) group

We next evaluated the effects of resveratrol on CBNPs-induced epithelial inflammation. Results indicated resveratrol not only decreased iNOS expression (Fig. 7a) and NO production (Fig. 7b), but also decreased COX-2 expression (Fig. 7c) and PGE_2_ production (Fig. 7d) in CBNPs-treated A549 cells.

### Resveratrol reduce CBNPs-induced PKC-α/Nox2/ROS activation in A549 cells

**Fig. 8:**
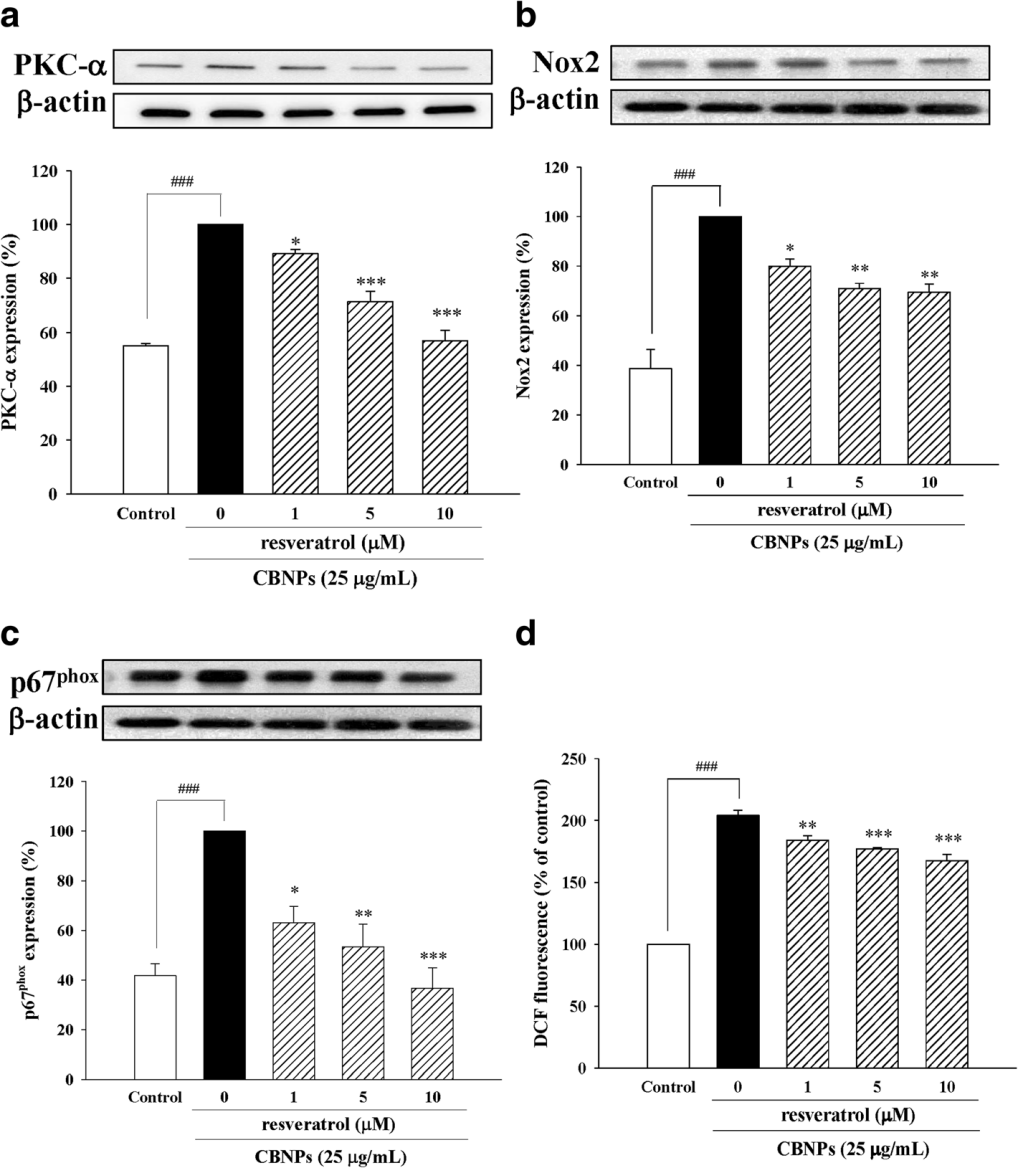
Effects of resveratrol on PKC-α expression (**a**), Nox2 expression (**b**), p67^phox^ membrane translocation (**c**), and ROS production (**d**) in CBNPs-treated A549 cells. Cells were pretreated with resveratrol for 1 h before addition of CBNPs (25 μg/mL) for 24 h. ROS production was measured using H_2_DCF-DA staining and detected the DCF fluorescence by flow cytometer. Bars represent the mean ± S.E.M. from six independent experiments. ^*###*^*P* < 0.001 vs. control group (without any treatment). ^*^P < 0.05, ^**^P < 0.01, ^***^P < 0.001 vs. CBNPs (25 μg/mL) group

We further investigated the protective effects of resveratrol on CBNPs-induced PKC-α expression, Nox2 activation and ROS production on A549 cells. Resveratrol attenuated PKC-α expression (Fig. 8a), Nox2 expression (Fig. 8b), p67^phox^ membrane translocation (Fig. 8c), and ROS production (Fig. 8d) induced by CBNPs.

## Discussion

The CBNPs are common air pollutants that are widely used in various commercial products as nano materials. CBNPs can readily suspend in the ambient atmosphere and cause airborne health issues when inhaled by humans due to their characteristically extreme small size, slight weight, and poor solubility. Bio-safety studies to provide data on CBNPs exposure are limited. Therefore, studies on mechanisms induced by CBNPs in human target cells would be helpful in preventing, identifying, and even treating the potential disorders caused by such particle exposure. The results generated by this study showed that PKC-α/Nox-mediated inflammation and oxidative stress contributed to CBNPs-induced toxicity in A549 cells.

Many studies have performed research on the cytotoxic effects of CBNPs [13, 32, 33]. A study on bronchial epithelial cells suggested that CBNPs induce ROS production, mitochondrial membrane potential loss, pro-apoptotic Bax activation, cytochrome *c* release, and cell apoptosis [13]. The present results supported the previous study [13] that CBNPs caused mitochondrial membrane potential loss, nuclear condensation and cell death.

Inflammation and oxidative stress are closely associated with many pulmonary diseases [34, 35]. Increasing evidence shows that ROS production plays a major role in inflammatory processes [36, 37]. It is reported that CBNPs induce inflammatory response by significantly inducing ROS production, IL-6 expression, and NFκB signaling, leading to COX-2 and TNF-α expression in RAW264.7 cells [14]. Moreover, NAC, a well-known ROS scavenger, attenuates CBNPs-induced NFκB [38], suggesting that ROS production plays a critical role in CBNPs-induced inflammation. The results obtained in this topic further demonstrated that PKC-α inhibitor significantly inhibited CBNPs-induced inflammatory responses via decreasing ROS production and leading to downregulation of NO and PGE_2_ in A549 cells, suggesting the important role of PKC-α in CBNPs-induced inflammatory pathways.

Nox activation plays an important role in ROS-mediated lung inflammation [39]. Our results demonstrated that CBNPs increased Nox2 expression and translocated its cytosolic subunit p67^phox^ to the membrane in A549 cells. Nox inhibitor (DPI) has been reported for its protective effects against CBNPs via inhibiting ROS production [12]. Here, we further demonstrated that DPI not only inhibited ROS, but also decreased CBNPs-induced inflammatory factors iNOS/NO and COX-2/PGE_2_ in A549 cells. Moreover, the present results showed that pretreatment of L-NAME or NS-398 could not significantly decrease CBNPs-induced Nox2 expression, suggesting CBNPs-induced Nox2 expression is not mediated via upregulation of iNOS and COX-2.

PKC plays a key role in different cellular signal transduction pathways [40]. It has been suggested as a master regulator of Nox activation [40, 41]. Activation of PKC-α, a Ca^2+^-dependent PKC, increases ET-1-induced COX-2/PGE_2_ in mouse brain microvascular endothelial cells [42], and increases LPS-induced iNOS/NO in alveolar epithelial cells [43]. A previous study demonstrated that CBNPs might cause intracellular Ca^2+^ increase in MM6 human monocytic cells [44]. The present results also showed that verapamil (L-type calcium channel blocker) and MK-801 (NMDA receptor antagonist) inhibit CBNPs-induced PKC-α activation. Therefore, we focused on investigating the role of PKC-α in CBNPs-induced inflammation in A549 cells. Our results demonstrated CBNPs-induced production of NO, PGE_2_, and ROS could be attenuated by Gö6976 (PKC-α inhibitor) pretreatment, suggesting that PKC-α might play a role in CBNPs-induced inflammation and oxidative stress. Moreover, the role of PKC-α in Nox-derived ROS production in pulmonary artery smooth muscle cells has been reported [45].

Therefore, we further evaluated the crosstalk between PKC-α and Nox in CBNPs-treated A549 cells. Our results indicated that inhibition of PKC-α by verapamil, MK801, and Gö6976 could inhibit CBNPs-induced Nox2 activation. However, inhibition of Nox by DPI and apocynin could not inhibit CBNPs-induced PKC-α activation. Taken together, PKC-α-mediated Nox activation contributed to CBNPs-induced inflammation and oxidative stress.

Resveratrol is a natural antioxidant in red wine and possesses a wide range of pharmacological anti-inflammatory, antioxidative, and anti-apoptotic properties, et al. [25, 46–48]. Resveratrol has previously been reported to repress oxidative and inflammatory lung injury against cigarette smoke exposure in vivo [49], and cigarette smoke is known as one of the major source of CBNPs [50]. Moreover, the present study reveals the important role of PKC-α/Nox in CBNPs-mediated inflammation and oxidative stress. Resveratrol has been reported to attenuate PMA-induced oxidative burst in isolated human neutrophils via PKC-α activation [51]; additionally, resveratrol decreases high glucose-induced endothelial apoptosis via Nox/ROS inhibition [52]. Recently, resveratrol is suggested to inhibit particulate matter-induced COX-2/PGE_2_ production in human fibroblast-like synoviocytes via attenuation of Nox/ROS/NF-κB [53]. However, the potential benefits of resveratrol on CBNPs-induced cytotoxicity have not yet been investigated. The present study further demonstrated resveratrol protected A549 cells against CBNPs-induced cytotoxicity. Moreover, resveratrol attenuated CBNPs-induced expression of inflammatory factors (iNOS/NO, COX-2/PGE_2_), and also inhibited Nox/ROS pathway in CBNPs-treated A549 cells. These results also suggest that fruits and food products containing resveratrol might provide health benefits to prevent NPs-induced inflammation.

## Conclusions

In the present study, we demonstrated the important role of PKC-α and Nox on CBNPs-induced inflammation and oxidative stress in human epithelial cells and reveal the potential protective effects of resveratrol against CBNPs-induced cytotoxicity, suggesting drug or dietary products providing PKC-α/Nox inhibitory effects might inhibit NPs-induced lung inflammation.

## Abbreviations

CBNPs: Carbon black nanoparticles
COX-2: Cyclooxygenase-2
iNOS: Inducible nitric oxide synthase
LDH: Lactate dehydrogenase
MTT: 3-(4,5-dimethylthiazol- 2-yl)-2,5-diphenyltetrazolium bromide
NO: Nitric oxide
Nox: NADPH oxidase
PGE_2_: Prostaglandin E_2_
PKC-α: Protein kinase C-α
ROS: Reactive oxidase species
ΔΨm: Mitochondrial membrane potential
